# Production of Spinocerebellar Ataxia Type 3 Model Mice by Intravenous Injection of AAV-PHP.B Vectors

**DOI:** 10.3390/ijms25137205

**Published:** 2024-06-29

**Authors:** Ayumu Konno, Yoichiro Shinohara, Hirokazu Hirai

**Affiliations:** 1 Department of Neurophysiology & Neural Repair, Gunma University Graduate School of Medicine, Maebashi 371-8511, Gunma, Japan; konnoa@gunma-u.ac.jp (A.K.); shinohara@gunma-u.ac.jp (Y.S.); 2 Viral Vector Core, Gunma University, Initiative for Advanced Research, Maebashi 371-8511, Gunma, Japan; 3 Department of Ophthalmology, Gunma University Graduate School of Medicine, Maebashi 371-8511, Gunma, Japan

**Keywords:** adeno-associated virus, AAV, AAV-PHP.B, Spinocerebellar ataxia type 3, SCA3, Machado–Joseph disease, Purkinje cell, nuclear inclusion bodies

## Abstract

We aimed to produce a mouse model of spinocerebellar ataxia type 3 (SCA3) using the mouse blood–brain barrier (BBB)-penetrating adeno-associated virus (AAV)-PHP.B. Four-to-five-week-old C57BL/6 mice received injections of high-dose (2.0 × 10^11^ vg/mouse) or low-dose (5.0 × 10^10^ vg/mouse) AAV-PHP.B encoding a SCA3 causative gene containing abnormally long 89 CAG repeats [ATXN3(Q89)] under the control of the ubiquitous chicken β-actin hybrid (CBh) promoter. Control mice received high doses of AAV-PHP.B encoding ATXN3 with non-pathogenic 15 CAG repeats [ATXN3(Q15)] or phosphate-buffered saline (PBS) alone. More than half of the mice injected with high doses of AAV-PHP.B encoding ATXN3(Q89) died within 4 weeks after the injection. No mice in other groups died during the 12-week observation period. Mice injected with low doses of AAV-PHP.B encoding ATXN3(Q89) exhibited progressive motor uncoordination starting 4 weeks and a shorter stride in footprint analysis performed at 12 weeks post-AAV injection. Immunohistochemistry showed thinning of the molecular layer and the formation of nuclear inclusions in Purkinje cells from mice injected with low doses of AAV-PHP.B encoding ATXN3(Q89). Moreover, ATXN3(Q89) expression significantly reduced the number of large projection neurons in the cerebellar nuclei to one third of that observed in mice expressing ATXN3(Q15). This AAV-based approach is superior to conventional methods in that the required number of model mice can be created simply by injecting AAV, and the expression levels of the responsible gene can be adjusted by changing the amount of AAV injected. Moreover, this method may be applied to produce SCA3 models in non-human primates.

## 1. Introduction

Spinocerebellar ataxia type 3 (SCA3), an autosomal dominant neurodegenerative disease, is caused by the abnormal expansion of a CAG repeat in *ATXN3* [1]. The mutant gene expresses through the development; however, the majority of the patients begin to develop symptoms in adulthood [2,3]. Since the mutant protein is degraded by the cellular quality control system such as the ubiquitin proteasome pathway and autophagy [2,3], one hypothesis to explain the late onset is deterioration of the intracellular degradation system associated with aging, leading to an accumulation of mutant protein in neurons, which compromises neuronal function.

To date, many SCA3 model mice, including transgenic mice and knock-in mice, have been developed [4]. Transgenic model mice are generated by the injection of linearized DNA consisting of a promoter and a mutant gene responsible for the SCA into fertilized eggs, which are then returned to surrogate mother mice. Since copy number and insertion sites in the mouse genome are random, it is difficult to obtain model mice with appropriate levels of transgene expression: excess levels of mutant gene expression cause significant developmental defects, whereas low expression does not result in abnormal phenotypes. Meanwhile, knock-in mice are generated by replacing the corresponding genetic locus with an abnormal gene, and they likely recapitulate the expression levels, expression cell types and timing of expression. However, knock-in mice exhibit only faint abnormal phenotypes after their aging, which may be because the abnormal gene expression is regulated by the mouse genome. Both transgenic and knock-in mouse production require wide breeding space, effort, and expense to maintain multiple lines.

An alternative approach is viral vector-mediated expression of the mutant gene to brain regions susceptible to the SCA3. A previous study injected lentiviral vectors encoding ATXN3(Q72) to the three-week-old mouse cerebellum [5], while another study injected adeno-associated virus (AAV) vectors encoding ATXN3(Q84) to neonatal mouse cerebellum [6]. These mice exhibited abnormal phenotypes seen in SCA3 patients, including motor uncoordination and nuclear inclusions in Purkinje cells. Compared with conventional gene-modified mice, the viral vector-mediated expression of a disease-causing gene in mice has the following advantages. (1) It is possible to control the timing of transgene expression simply by changing the age of the mice for viral injection. (2) By using adult wild-type mice for virus injections, it is possible to eliminate developmental defects caused by nonphysiological overexpression of the transgene. (3) Levels of transgene expression can be manipulated by changing the dose of AAV injected. (4) Disease-causing genes having CAG repeats of different length and/or different mutations can be tested by simply changing transgenes accommodated in the viral vector followed by its injection to mice. However, brain parenchymal injections result in locally restricted and heterogeneous transgene expression. Expression levels are high around the injection needle and decrease with increasing distance. To overcome the drawback of direct tissue injection, we used the mouse blood–brain barrier (BBB)-penetrating AAV-PHP.B [7]. The four- to five-week-old mice received intravenous injections of AAV-PHP.B encoding ATXN3(Q89), and the resultant mice were evaluated by behavioral tests and immunohistochemistry.

## 2. Results

### 2.1. Efficient and Broad Transgene Expression in the Brain by Intravenous Injection of AAV-PHP.B

**Figure 1:**
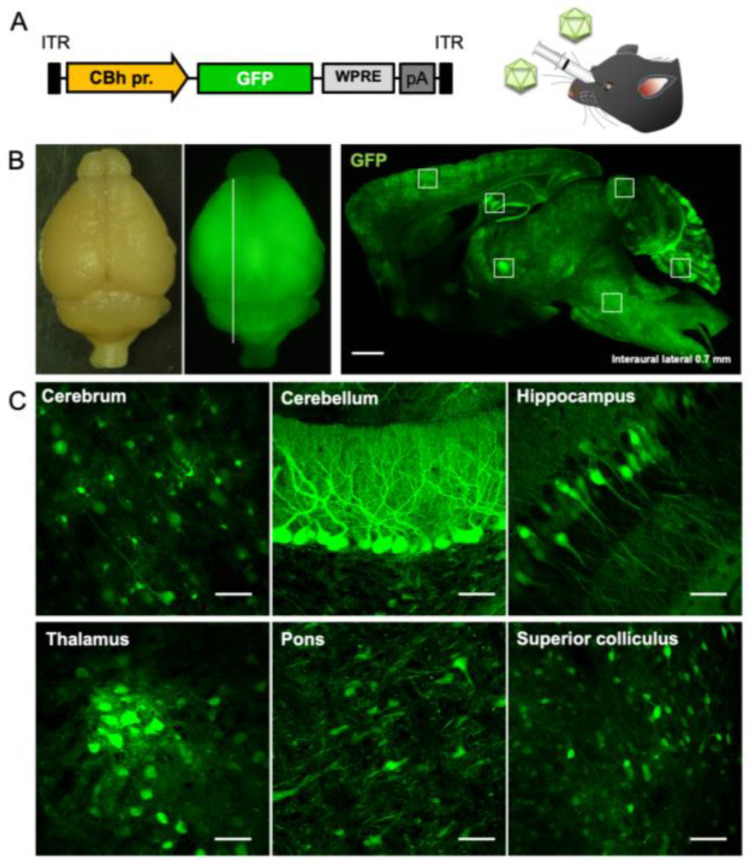
Broad and efficient transgene expression in the mouse brain by intravenous injection of AAV-PHP.B. (**A**) Expression cassette of the AAV genome. The ubiquitous CBh promoter drives GFP expression. WPRE and polyadenylation signal (pA) sequence were placed downstream of the GFP gene. (**B**) Bright field and fluorescent stereo microscopy of the whole brain (left). Right fluorescent image was obtained by cutting at the white line drawn on the left side of the brain. Scale bar, 1 mm. (**C**) Efficient GFP expression in different regions of the brain by intravenously injected AAV-PHP.B. Square regions on the sagittal section of the brain (right image in (**B**)) are enlarged. Scale bars; 50 µm.

Initially, we validated the transgene expression profile when AAV-PHP.B was intravenously injected to mice. Five-week-old C57BL/6 mice received injections of AAV-PHP.B encoding GFP by the ubiquitous CBh promoter (1.0 × 10^12^ vg/mouse) through the orbital plexus (Figure 1A). Mice were sacrificed 3 weeks after the viral injection. Bright field and fluorescent stereo microscopy of the whole brain and fluorescent microscopic examination of the sagittal section showed GFP expression throughout the brain (Figure 1B). Magnified images confirmed that the intravenous injection of AAV-PHP.B achieved efficient GFP expression in various brain regions including cerebral cortex, cerebellum, hippocampus, thalamus, pons, and superior colliculus (Figure 1C). Variable GFP expression is likely due to (1) different BBB permeability of AAV-PHP.B capsid through brain microvascular endothelial cells, depending on brain regions, (2) varied tropism of AAV-PHP.B capsid toward cell types in different brain regions [7], and (3) inhomogeneous CBh promoter activity in different cell types in different brain regions. These factors are overall similar among individuals of the same inbred strain [8], suggesting similar transgene expression profiles upon the intravenous injection of AAV-PHP.B in the C57BL/6 mouse CNS. Based on the GFP expression profile in the CNS and the underlying considerations, we planned to generate SCA3 model mice by delivering the mutant gene responsible for SCA3.

### 2.2. Early Death in Mice That Received High Doses of AAV Encoding ATXN3 with Abnormally Expanded Polyglutamine Tract

**Figure 2:**
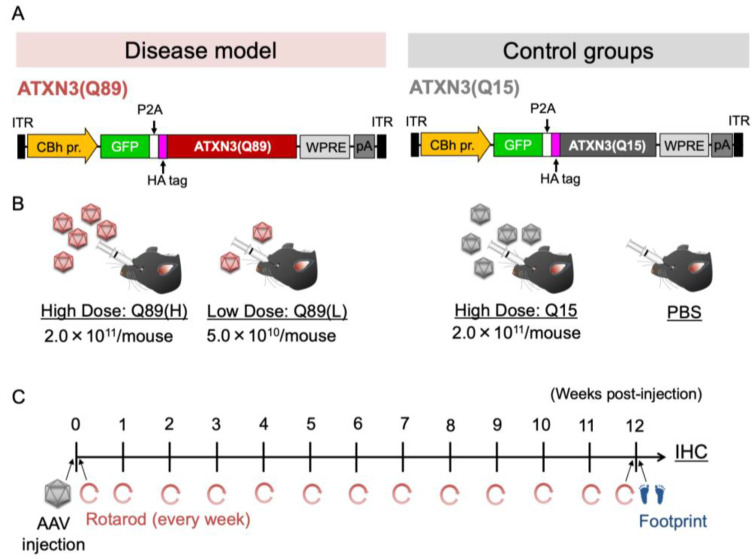
Schema showing the experimental protocol. (**A**) Expression cassettes of the AAV genomes. The ubiquitous CBh promoter drives the expression of GFP-P2A-ATXN3(Q89) or -ATXN3(Q15). HA-tag was attached at the 5′-terminal of ATXN3 for immunolabeling the expressed ATXN3 protein by anti-HA antibody. (**B**) Schema showing experimental groups. Four-five-week wild-type mice were intravenously injected with high doses of BBB-permeable AAV-PHP.B encoding ATXN3(Q89), low-dose ATXN3(Q89) or high-dose ATXN3(Q15), or with PBS. (**C**) Diagram depicting the experimental protocol. A rotarod test was completed just before and every week up to 12 weeks after the AAV injection. After footprint analysis at 12 weeks, mice were sacrificed and subjected to immunohistochemistry (IHC).

Four–five-week-old C57BL/6 mice received injections of high-dose (2.0 × 10^11^ vg/mouse) or low-dose (5.0 × 10^10^ vg/mouse) AAV-PHP.B vectors encoding GFP-P2A-HA-tagged ATXN3 with abnormally expanded 89 polyglutamine tract [ATXN3(Q89)] under the control of the ubiquitous CBh promoter through the orbital plexus (Disease model; Figure 2A,B). We prepared two control mouse groups. One group received an intravenous injection of high-dose AAV-PHP.B encoding ATXN3 carrying 15 glutamine chain [ATXN3(Q15)] instead of ATXN(Q89). Another group received an injection of PBS alone. Each mouse was assessed for its behavioral performance on the time schedule shown in Figure 2C.

**Figure 3:**
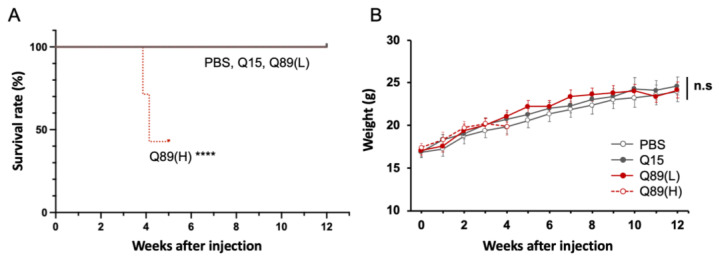
Effects of AAV-mediated mutant ATXN3 expression on the survival and gaining body weight. (**A**) Survival curves of mice injected with high doses of AAV-PHP.B encoding ATXN3(Q89) or ATXN3(Q15), or low doses of AAV-PHP.B encoding ATXN3(Q89) or with PBS. **** *p* < 0.0001 by log-rank (Mantel–Cox) test. (**B**) Graph showing weight gain of mice. Measurement of mice injected with high doses of AAV-PHP.B encoding ATXN3(Q89) was stopped 4 weeks after injection as more than half of the mice died. n.s., not significant by one-way analysis of variance (ANOVA) followed by Tukey’s post hoc test at each time point.

All groups of mice appeared normal in their home cages during a couple of weeks after the viral injection; however, mice that received high doses of AAV-PHP.B encoding ATXN3(Q89) began to die around 3 weeks post-AAV injection (Figure 3A). Other AAV-treated mouse groups did not die during the 12-week observation period for and gained weight similar to the PBS-treated mouse group (Figure 3B).

### 2.3. Motor Defects of Mice That Received AAV Encoding ATXN3(Q89)

**Figure 4:**
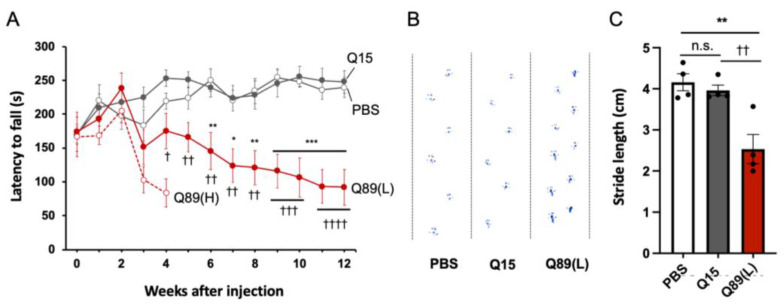
Progressive motor deficits of mice injected with low doses of AAV-PHP.B encoding ATXN3(Q89). (**A**) Time course of rotarod performance of mice injected with AAV or PBS. Rotarod test was completed just before and every week post-injection. Mice were placed on a rod while accelerating from 4 to 40 rpm in 5 min, and the time it took for the mice to fall off the rod was measured. The average latency to fall of 4 trials was recorded. *n* = 7 [PBS, Q89(H)] or 8 [Q15, Q89(L)] mice, respectively. * *p* < 0.05, ** *p* < 0.01, *** *p* < 0.001 [vs. PBS], † *p* < 0.05, †† *p* < 0.01, ††† *p* < 0.001, †††† *p* < 0.0001 [vs. Q15(H)] by one-way analysis of variance (ANOVA) followed by Tukey’s post hoc test at each time point. (**B**,**C**) Footprint analysis performed 12 weeks after AAV injection. Mice with their hind limbs painted with a blue dye walked on a white paper for 30 cm (**B**). The average stride length was measured, excluding the beginning and end of the footprints (**C**). *n* = 4 mice, respectively. **, †† *p* < 0.01 by one-way analysis of variance (ANOVA) followed by Tukey’s post hoc test. n.s., not significant.

The effect of mutant ATXN3 expression on mouse behavior was assessed just before and every week after the AAV injection by rotarod test (Figure 2C). All groups of mice could stay on the rotating rod for around 170 s at the first trial, which was completed just before the viral injection (Figure 4A). The performance of control mice that received PBS alone and those expressing ATXN3(Q15) improved gradually and plateaued 4–6 weeks after AAV injection. In contrast, mice treated with high-dose AAV encoding ATXN3(Q89) showed clear ataxic gating in their home cage and significantly poorer performance on the rotarod test at 3 weeks post-viral injection. Four weeks after the viral injection, symptoms such as sensory hypersensitivity and excited state appeared, and more than half of the mice died (Figure 3A). Therefore, the further behavioral assessment of mice injected with high doses of AAV-PHP.B encoding ATXN3(Q89) was discontinued.

Mice treated with low doses of AAV encoding ATXN3(Q89) gained weight similarly with mice treated with PBS alone (Figure 3B). Motor defects in mice expressing low doses of ATXN3(Q89) were first detected by the rotarod test at 4 weeks after the viral injection and gradually progressed (Figure 4A), although the behavior in their home cages was largely indistinguishable from PBS-injected control mice until 6 to 7 weeks after viral injection. Some mice expressing low doses of ATXN3(Q89) began to exhibit slight ataxic behavior in the home cage from the latter half of the experimental schedule (about after 8 weeks), but no other clear phenotypes could be confirmed during the observation period.

Motor function was further assessed by footprint analysis after the final rotarod test at 12 weeks post-viral injection. The stride length of mice injected with high doses of AAV-PHP.B encoding ATXN3(Q15) was comparable to that of mice that received PBS alone (Figure 4B,C). In contrast, the stride length of mice injected with low doses of AAV-PHP.B encoding ATXN3(Q89) was significantly smaller compared with other two control mouse groups.

### 2.4. Significantly Reduced Thickness of the Molecular Layer of the Cerebellar Cortex in Mice Injected with AAV-PHP.B Encoding ATXN3(Q89)

**Figure 5:**
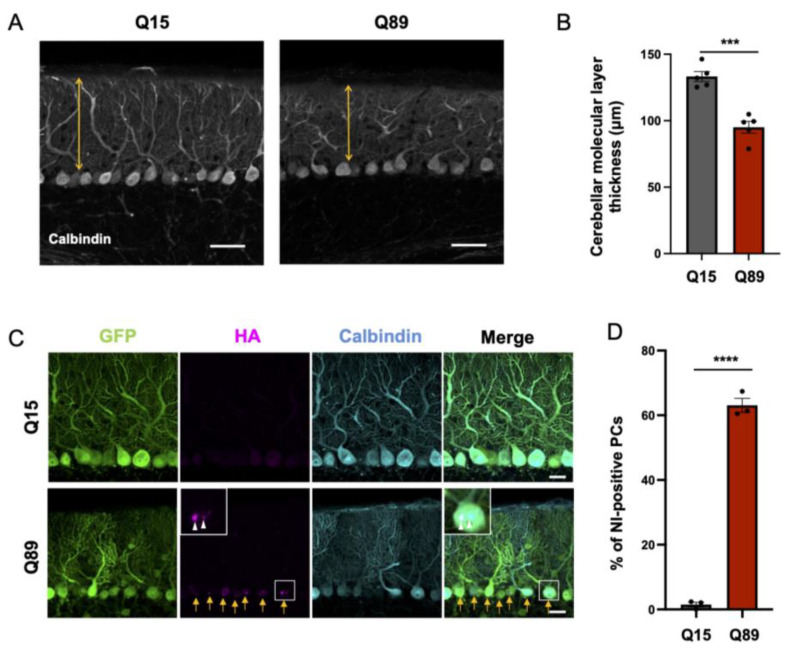
Dendrite atrophy and nuclear inclusion body formation in Purkinje cells. (**A**) Decreased molecular layer thickness in the cerebellar cortex of mice injected with low doses of AAV-PHP.B encoding ATXN3(Q89). Length depicted with double-headed arrows at the center of lobule 6 were measured using 4 to 6 sections per mouse. The mean value was used as a cerebellar molecular layer thickness of each mouse. Scale bars; 50 µm. (**B**) Quantitative analysis of the molecular layer thickness showing significantly smaller thickness in mice injected with low doses of AAV-PHP.B encoding ATXN3(Q89) compared with mice injected with high doses of AAV-PHP.B encoding ATXN3(Q15). n = 5 mice, respectively. *** *p* < 0.001 by unpaired *t*-test. (**C**) Nuclear inclusion body formation in Purkinje cells of mice injected with AAV-PHP.B injected with low doses of ATXN3(Q89). Cerebellar sections were double immunolabeled for HA (magenta) and calbindin (blue). Arrows indicate Purkinje cells contained nuclear inclusions. White arrowheads in the enlarged insert indicate nuclear inclusion body. Scale bars; 20 µm. (**D**) Graph showing quantitative analysis of ratios of nuclear inclusion (NI)-positive Purkinje cells (PC) to total Purkinje cells. n = 3 mice, respectively. **** *p* < 0.0001 by unpaired *t*-test.

Previous studies showed reduced thickness of the molecular layer of the cerebellar cortex in SCA3 model mice [9,10], indicating an atrophy of Purkinje cell dendrites. We examined whether a similar shrinkage of Purkinje cell dendrites was observed also in SCA3 model mice that received low doses of AAV vectors encoding ATXN3(Q89). Twelve weeks after the viral injection, the molecular layer thickness, measured at the center of lobule 6 using 4 to 6 sections per mouse, was significantly thinner in mice expressing ATXN3(Q89) than in those expressing ATXN3(Q15) (Figure 5A,B).

### 2.5. Nuclear Inclusion Body Formation in Purkinje Cells of Mice Expressing ATXN3(Q89)

Purkinje cells of conventional SCA3 model mice form mutant protein-containing nuclear inclusion, which is a hallmark of neurodegenerative diseases [11,12]. Then, we explored whether similar nuclear inclusions were observed in Purkinje cells of mice that received injections of AAV vectors encoding ATXN3. AAV-derived ATXN3 protein was visualized by immunolabeling HA-tag attached at the N-terminus of ATXN3. Confocal microscopy examination showed that more than 60% of Purkinje cells contained nuclear inclusions of mutant protein in mice injected with low doses of AAV-PHP.B encoding ATXN3(Q89), which is in contrast to the absence of the nuclear inclusions in Purkinje cells of mice injected with high doses of AAV-PHP.B encoding ATXN3(Q15) (Figure 5C,D).

### 2.6. Significant Decrease in Large Projection Neurons in the Deep Cerebellar Nuclei of Mice Injected with Low Doses of AAV-PHP.B Encoding ATXN3(Q89)

**Figure 6:**
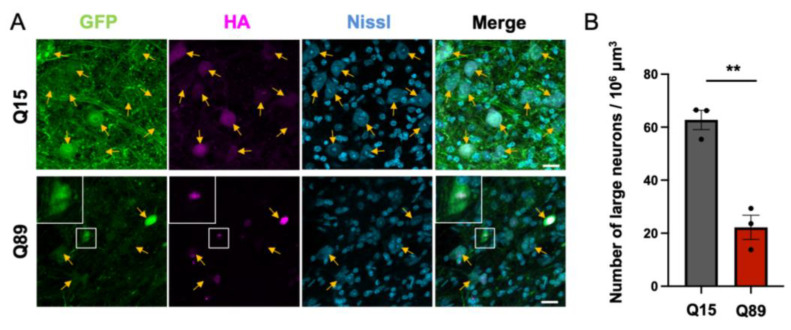
Significant loss of large projection neurons in the cerebellar nuclei of mice expressing ATXN3(Q89). (**A**) Fluorescent images of the cerebellar nuclei double immunolabeled for HA (magenta) and Nissl (blue). Arrows indicate large projection neurons. Scale bar; 20 µm. (**B**) Graph showing quantitative analysis of number of large neurons per 10^6^ cubic micrometers of cerebellar nucleus. n = 3 mice, respectively. ** *p* < 0.01 by unpaired *t*-test.

Purkinje cells are sole output neurons from the cerebellar cortex, and their axons project to the deep cerebellar nuclei. Previous studies showed that Purkinje cell and deep cerebellar nuclei are the targets of the degeneration in SCA3 patients: deep cerebellar nuclei underwent a marked to serious neuronal loss in SCA3 patients [13,14]. Therefore, we examined whether the deep cerebellar nuclei were impaired in mice injected with AAV encoding ATXN3(Q89). Neurons in the deep cerebellar nuclei were labeled with the fluorescent Nissl stain NeuroTrace [15], and AAV-derived ATXN3 were immunolabeled for HA. GFP and HA-tagged ATXN3 were observed in large projection neurons (arrows in Figure 6A). HA immunolabeling was aggregated in the nucleus of the projection neurons in mice injected with low doses of AAV-PHP.B encoding ATXN3(Q89), whereas it was diffusely distributed in the cytoplasm and nucleus in mice injected with high doses of AAV-PHP.B encoding ATXN3(Q15). Of note, we noticed that the number of large neurons was reduced in mice expressing ATXN3(Q89) compared with those in mice expressing ATXN3(Q15) (Figure 6A). Quantitative analysis showed that the number of neurons with cell body diameters >10 μm in the cerebellar nuclei of mice expressing ATXN3(Q89) was reduced to approximately one third of that in the same region of mice expressing ATXN3(Q15) (Figure 6B). These results suggest that ATXN3(Q89) expression significantly reduced large projection neurons in the deep cerebellar nuclei, as large neurons with cell body diameters >10 μm in the cerebellar nuclei cover the majority of glutamatergic projection neurons [16].

## 3. Discussion

In this study, we aimed to produce SCA3 model mice through the intravenous injection of mouse BBB-penetrating AAV vectors. Mice injected with AAV encoding ATXN3(Q89), but not mice injected with AAV encoding ATXN3(Q15), exhibited progressive motor deficits, dendrite atrophy, and the formation of nuclear inclusions in Purkinje cells and neuronal loss in the cerebellar nuclei.

AAV-PHP.B is a BBB-penetrating capsid variant first reported by Gradinaru’s group [7]. AAV-PHP.B has 40-times higher capacity of crossing mouse BBB than AAV9 [7]. Subsequently, the same group reported a capsid mutant AAV-PHP.eB with higher BBB penetration ability. Using AAV-PHP.eB, which crosses the BBB more efficiently than AAV-PHP.B, can reduce the injection volume for creating mouse models. One thing to note is that both PHP.B and PHP.eB infect neurons far more than astrocytes [7,17,18]. In contrast, AAV-F [19], a different BBB-penetrating capsid, infects neurons and astrocytes without bias [20]. Thus, AAV-F is preferable in cases targeting astrocytes as well as neurons.

Like its parent AAV9, AAV-PHP.B is highly tropic to hepatocytes. When administered intravenously at high doses, AAV-PHP.B encoding ATXN3(Q89) by the ubiquitous CBh promoter can cause severe liver dysfunction and coagulation disorders, as reported in Cynomolgus Macaques [21,22], which may explain a cause of the early mortality of animals that received high doses of AAV-PHP.B encoding ATXN3(Q89). Adverse influences by off-target tissue transduction may be attenuated by using a capsid less tropic to hepatocytes, a capsid mutant more efficiently crossing the BBB to reduce the injection dose and/or brain cell type-specific promoters to restrict a transgene expression to brain cells.

The model mice produced by intravenously injecting AAV can be used for a variety of purposes, including screening for effective drugs against target diseases. However, care must be taken when using them for the preclinical testing of gene therapy. Most BBB-penetrating capsid variants are derived from AAV9, including AAV-PHP.B, AAV-PHP.eB, and AAV-F. These capsid mutants can be used only once, because the first injection triggers the production of neutralizing antibodies against AAV9, which cross-reacts with AAV9 variants as well. Therefore, for the second intravenous injection of AAV, it is indispensable to use BBB-penetrating capsid variants derived from a different serotype that does not cross-react with AAV9 capsids, such as an AAV2-derived BR1N [23].

The greatest advantage of producing animal models using AAV vectors is that this method can also be applied to non-human primates (NHPs) such as common marmosets and macaque monkeys. In these cases, capsid variants that can cross the NHP BBB should be used, since AAV-PHP.B, AAV-PHP.eB, and AAV-F can only cross the mouse BBB [8,24,25]. Recent studies have developed AAV capsid mutants that efficiently penetrate the BBB of multiple primate species: marmoset, rhesus macaque, green monkey [26,27] and human [28]. Such capsid variants that efficiently penetrate the primate BBB will facilitate the production of neurodegenerative disease models of NHPs including SCA3 and will contribute to develop therapeutics against the neurodegenerative diseases.

## 4. Materials and Methods

### 4.1. Animals

Wild-type C57BL/6 mice between 4 and 5 weeks of age were bred at the Gunma University Bioresource Center (Maebashi, Gunma, Japan) for use in this study. Mice were maintained on a 12 h light/dark cycle and had access to food and water ad libitum. A sex-balanced group of mice was used for this study. All animal care and treatment procedures were performed in accordance with the Japanese Act on the Welfare and Management of Animals and the Guidelines for Proper Conduct of Animal Experiments issued by the Science Council of Japan. The experimental protocol was approved by the Institutional Committee of Gunma University (No. 17-026; 17-034). All efforts were made to minimize suffering and to reduce the number of animals used. A humane endpoint during the animal experiments was defined as the following indicators: severe pain, severe distress, suffering or impending death, at which conditions, mice were humanely euthanized by isoflurane inhalation or cervical dislocation.

### 4.2. AAV Vector Preparation

The pAAV expression plasmid comprises a ubiquitous chicken ß-actin hybrid (CBh) promoter [29], woodchuck hepatitis virus post-transcriptional regulatory element (WPRE), and a simian virus 40 (SV40) polyadenylation signal sequence. The transgenes, GFP, GFP-P2A-HA-ATXN3(Q15), or GFP-P2A-HA-ATXN3(Q89), were incorporated between the CBh promoter and GFP at AgeI and NotI-digested sites of pAAV vectors. The CAG repeat length in human ATXN3 cDNA was artificially expanded to 89 repeats using PCR-based trinucleotide repeat expansion [30]. The Rep/Cap plasmid for PHP.B [7], pAAV-PHP.B, was constructed by replacing the wild-type fragment between the BsiWI and PmeI sites of pAAV2/9 (kindly provided by Dr. J. Wilson from University of Pennsylvania) with the mutant capsid gene fragment, containing the partial PHP.B VP1 gene (GenBank KU056473).

Recombinant single-strand AAV-PHP.B vectors were produced by the ultracentrifugation method as reported previously [31]. Briefly, three plasmids, the expression plasmid pAAV, pHelper (Agilent Technologies, Santa Clara, CA, USA), and pAAV-PHP.B, were co-transfected using polyethylenimine (24765-1; Polysciences, Inc., Warrington, PA, USA) into HEK293T cells (HCL4517; Thermo Fisher Scientific, Waltham, MA, USA) cultured in Dulbecco’s modified Eagle’s medium (DMEM; D5796-500Ml; Sigma-Aldrich, St. Louis, MO, USA) supplemented with 8% fetal bovine serum (Sigma-Aldrich). Viral particles were harvested from the culture medium 6 days after transfection and concentrated by precipitation with 8% polyethylene glycol 8000 (P5413; Sigma-Aldrich) and 500 mM sodium chloride. The precipitated AAV particles were resuspended in Dulbecco’s phosphate-buffered saline (D-PBS) and purified with iodixanol (Optiprep; AXS-1114542-250ML; Alere Technologies, Oslo, Norway) linear gradient ultracentrifugation. The viral solution was further concentrated and formulated with D-PBS using a Vivaspin 20 column (VS2041 or VS2042; Sartorius, Göttingen, Germany).

The genomic titers of the purified AAV-PHP.B vectors were determined by quantitative real-time PCR using THUNDERBIRD™ SYBR qPCR Mix (Toyobo, Osaka, Japan) with the primers 5′-CTGTTGGGCACTGACAATTC-3′ and 5′-GAAGGGACGTAGCAGAAGGA-3′, targeting the WPRE sequence. The expression plasmid vectors were used as standards.

### 4.3. AAV Injection through Retro-Orbital Sinus

After deep anesthesia via intraperitoneal injection of ketamine (100 mg/kg body weight) and xylazine (10 mg/kg body weight), 100 µL of the AAV vector preparation was intravenously injected into the retro-orbital sinus of mice over a period of 1 min, using a 1 mL syringe attached to a 29-gauge needle (SS-10M2913A, TERUMO, Tokyo, Japan). The syringe was kept in place for 30 s following the injection. The same volume of PBS was injected intravenously as a control.

### 4.4. Behavioral Tests

The motor coordination ability for SCA3 model mice was evaluated using a rotarod test (MK-610A/RKZ, Muromachi Kikai, Tokyo, Japan) and footprint analysis. Mice were subjected to 4 trials separated by 30-minute intervals on the rod while accelerating from 4 to 40 rpm in 5 min. The rotarod tests were conducted at 1-3 days prior to AAV injection and every week up to 12 weeks after AAV injection. The average latency to fall of 4 trials was recorded.

The footprint analysis was performed 12 weeks after AAV injection. Mice with their hind limbs painted with a blue dye walked on a white paper for 30 cm. The average stride length was measured, excluding the beginning and end of the footprints.

### 4.5. Immunohistochemistry

After deep anesthesia via intraperitoneal injection of ketamine (100 mg/kg body weight) and xylazine (10 mg/kg body weight), mice were transcardially perfused with phosphate-buffered saline (PBS) (pH 7.4) and 4% paraformaldehyde in 0.1 M phosphate buffer (PB). The whole brains were immersed in 4% paraformaldehyde for 6 h at 4 °C and cut into 50 µm sagittal sections using a microtome (Leica VT1000 S; Leica Microsystems, Wetzlar, Germany). Free-floating sagittal brain sections were blocked with PBS containing 2% normal donkey serum, 0.1% Triton X-100, and 0.05% NaN_3_ (blocking solution) and were then incubated overnight at 4 °C in the primary antibodies. The primary antibodies used in this study were rabbit polyclonal anti-GFP (1:1000; Rb-Af2020; Frontier Institute, Hokkaido, Japan), rat monoclonal anti-HA (1:1000; clone 3F10; Roche, Mannheim, Germany), and mouse monoclonal anti-Calbindin D-28k (1:500; 300; Swant, Bellinzona, Switzerland). After washing for 3 times with PBS at room temperature, the slices were incubated in blocking solution for 4 h at room temperature with the secondary antibodies. The secondary antibodies used were Alexa Fluor 488 donkey anti-rabbit IgG (1:1000; Thermo Fisher Scientific, Waltham, MA, USA), Alexa Fluor 594 donkey anti-rat IgG (1:1000; Thermo Fisher Scientific), and Alexa Fluor 680 donkey anti-mouse IgG (1:500; Thermo Fisher Scientific). For Nissl staining, the slices were treated with NeuroTrace 640/660 (1:200; Thermo Fisher Scientific) in PBS for 1 h at room temperature. After washing 3 times with PBS at room temperature, slices were mounted on glass slides with ProLong Diamond antifade reagents (Thermo Fisher Scientific).

### 4.6. Imaging Analysis

Fluorescence images were acquired on a fluorescence microscope (VB-7010 or BZ-X700; Keyence, Osaka, Japan) or a confocal laser-scanning microscope (LSM 800; Carl Zeiss, Oberkochen, Germany). The GFP fluorescence images were captured using a confocal microscope with the same settings in each experimental group.

To determine the thickness of the cerebellar molecular layer, we measured the length at the center of lobule 6 because the efficiency of PHP.B infection in this region is stable and exhibits little variation among mice. Four to six slices were used for measurement, and the mean value was taken as the thickness of the molecular layer for each mouse.

To quantify the percentage of nuclear inclusion (NI)-positive Purkinje cells, we utilized double immunostained images with HA antibody and calbindin antibody (a marker for Purkinje cells). Since HA is fused with ATXN3, mutant ATXN3 aggregates can be detected as obvious dot-like HA-immunoreactive signals in the nucleus of calbindin-immunoreactive Purkinje cells (Figure 5C). At least 40 Purkinje cells were counted from each mouse, and Purkinje cells with one or more aggregates detected were defined as NI-positive Purkinje cells.

For counting the number of large projection neurons in the deep cerebellar nuclei, confocal laser-scanning images of the deep cerebellar nuclei stained with NeuroTrace 640/660 fluorescent Nissl stain (Thermo Fisher Scientific) were obtained by the projection of 16–20 serial sections in 1 µm intervals. The Nissl stain detect both large glutamatergic projection neurons and smaller interneurons. Because of the overlap, it is difficult to completely distinguish between the two cell types based on cell body diameter alone. We counted the number of neurons with a cell body diameter > 10 μm, since this criterion covers most of the large projection neurons, although it includes some interneurons [16]. The large neuron density in the deep cerebellar nuclei was normalized to a density per 1,000,000 µm^3^.

### 4.7. Data and Statistical Analysis

Statistical analyses were performed using GraphPad Prism 10 (GraphPad Software, San Diego, CA, USA). Significant differences were analyzed using Student’s *t*-test or one-way analysis of variance (ANOVA) followed by Tukey’s post hoc test. The survival rates were investigated using Kaplan–Meier curves and log-rank (Mantel-Cox) analysis. A *p*-value < 0.05 was considered statistically significant. All data were presented as the mean ± SEM. The data that support the findings of this study are available from the corresponding author upon reasonable request.

## Data Availability

The data that support the findings of this study are available from the corresponding author upon reasonable request.
